# Works Published by the Sydenham Society

**Published:** 1847-01

**Authors:** 


Works published by the Sydenham Society.

Art. XVI.—1. *An Anatomical Description of the Diseases of the Organs of Respiration.* By C. E. Hasse, m.d. Translated and edited by W. E. Swaine, m.d.—*London,* 1846. 8vo, pp. 400.

2. *The Seven Books of Paulus Ægineta.* Translated from the Greek with a Commentary. By Francis Adams. Vol. II.—*London,* 1846. 8vo, pp. 510.

3. *The Works of William Hewson, F.R.S.* Edited with an introduction and notes, by George Gulliver, f.r.s.—*London,* 1846. 8vo, pp. 360.

These three volumes, so beautifully printed and so elegantly got up, are the handsome return which theSydenham Society has made to each of its subscribers for his guinea of 1846, the lowest price of which, in the shops, would be nearer three guineas than two. And yet the beauty of these volumes is their least charm. We have made to ourselves a vow not to analyse any of the works of this Society, and for these reasons: our readers either have the books or they have them not: if they have them they do not require any account of them from us: if they have them not, we do them a service by thus lending our aid to compel them to join the Society and so to possess these volumes and others of like value. Of the works before us, we will merely say that *Hasse's* is a unique treatise on its important subject, much improved by the author's manuscript additions and excellently translated by Dr. Swaine; that the second volume of *Paulus* is no less curious than the first, and displays the same store of classic learning in its accomplished translator, Dr. Adams; and that the great original value of *Hewson's* works is here doubled by the commentaries or notes of Mr. Gulliver. This volume is, moreover, illustrated with all Hewson's plates, finely re-engraved in a reduced size, and has a charming portrait of the author as a frontispiece.

We are happy to learn that this excellent Society is very prosperous, having now considerably more than 2000 members. We trust the Council will proceed in their present course, giving us works of real value without regard to any stupid clamour that may be raised against them. It is their duty to do what they themselves think best for directing and improving the literary and scientific taste of their subscribers generally, not to cater to the unripe judgment and silly wants of the uninformed.

